# Concussions and Repercussions

**DOI:** 10.1371/journal.pmed.1002104

**Published:** 2016-08-23

**Authors:** Donald A. Redelmeier, Sheharyar Raza

**Affiliations:** 1 Department of Medicine, University of Toronto, Toronto, Ontario, Canada; 2 Evaluative Clinical Sciences Program, Sunnybrook Research Institute, Toronto, Ontario, Canada; 3 Institute for Clinical Evaluative Sciences, Toronto, Ontario, Canada; 4 Division of General Internal Medicine, Sunnybrook Health Sciences Centre, Toronto, Ontario, Canada; 5 Center for Leading Injury Prevention Practice Education & Research Toronto, Ontario, Canada

## Abstract

In their Perspective, Donald A. Redelmeier and Sheharyar Raza discuss the significance of Seena Fazel and colleagues' longitudinal study of traumatic brain injury (TBI)-associated outcomes.

Popular fiction spreads a myth that concussions leave no lasting damage. Consider, for example, the young, exuberant cartoon character Tintin who is knocked unconscious by dozens of events yet recovers fast and fully for the next adventures [1]. Blockbuster movies extend the myth, depicting heroic characters such as James Bond and Indiana Jones who bounce back easily from multiple concussions. In contrast, anecdotes of professional athletes and military veterans suggest that traumatic brain injury may lead to later depression, dementia, and lives that slowly unravel. No story, however, provides scientific evidence to inform the debate on whether concussions acquired in everyday circumstances contribute to adverse psychosocial outcomes [2].

In this issue of *PLOS Medicine*, Fazel and colleagues report a study of over a million children and adolescents diagnosed with a traumatic brain injury compared to their unaffected siblings [3]. The findings show a significant association of injury in childhood with poor psychosocial functioning in adulthood, as exemplified by a doubling in subsequent psychiatric hospitalizations years later. The study shows further worrisome associations with low educational achievement, welfare enrollment, and long-term disability pensions. These differences were particularly large for youths with severe brain injuries that occurred after 10 years of age and were followed by repeated events. The majority of cases studied, however, were in youths with a single mild head injury (hereafter called a concussion).

The study by Fazel and colleagues has limitations that justify cautious interpretation. Relative risks are not absolute risks since the baseline is modest and most individuals seem to recover fully; for example, the absolute increased incidence of psychiatric hospitalizations was about 4.7 per 100 patient-years in analyses comparing those with a concussion to those without a concussion (10.4 versus 5.7, *p* < 0.001). This increase equals a number-needed-to-harm of approximately 21 and is even more modest for other outcomes. This means that most individuals do not experience adverse outcomes. Of course, the median follow-up was only 8 years, the curves depicting adverse psychosocial events widened with time, and the absolute risks might worsen at more advanced ages.

A second limitation is that correlation does not prove causality since a concussion in childhood might merely mean a predisposition to psychological conditions destined to cause trouble later. In this study, however, the observed comparison to siblings adjusts for multiple unmeasured factors, including parental involvement and socioeconomic status. Moreover, the median age at injury was 13 years and at follow-up was 34 years, thereby indicating a two-decade separation between exposure and outcome. That’s a long time for a latent confounder to stay hidden from multivariable analysis. These data, therefore, comprise the strongest available long-term analysis of concussions in youths, since a randomized trial is unethical and animal experiments cannot examine psychosocial outcomes [4].

As common for a study of this size, the available data do not provide clinical details such as the direction of linear forces, rotational acceleration, impact location, and other biomechanics for any individual. The severity of injury is assessed crudely, with no information on the duration of unconsciousness, extent of amnesia, degree of disorientation, limitations in concentration, and other neurologic signs. Each person’s management is unique, and the study does not account for pediatric care, psychiatric intervention, rehabilitation therapy, or other interventions over time. The specific indicators of adulthood psychosocial functioning are reasonable but do not include career productivity, quality of life, or community engagement.

The study by Fazel and colleagues is mostly silent about the mechanisms of how concussions could cause adverse long-term psychosocial consequences. One theory is that blunt forces can damage hundreds of neurons and millions of synapses, thereby contributing to subsequent depression, irritability, impulsivity, or suicidal ideation [5]. A different mechanism might be that concussions result in only temporary impairments that interrupt school or social interactions and that therefore change a person’s life trajectory due to the missed opportunities [6]. A further consideration is that individuals misunderstand why they don’t recover rapidly, lose self-esteem, blame themselves as lazy, or become directly discouraged [7]. Untangling these theories awaits future research.

Another possible mechanism is that a concussion acts in concert by complicating the care of an underlying chronic disease, a nuance unexplored by Fazel and colleagues. Some youth, for example, might also have diabetes, asthma, epilepsy, sickle cell anemia, cystic fibrosis, or cerebral palsy. In each case, chronic disease care may be compromised by a concussion that interferes with self-management, so that subsequent disability progresses at an intensified pace [8]. The result, for example, may be that adult kidney failure is attributed to poor diabetes control without realizing the contribution from a remote concussion. This misattribution of causality may be particularly beguiling if nephrologists or other specialists do not elicit a past trauma history [9].

The main implication of Fazel and colleagues is to emphasize the importance of preventing concussions in youth. Some basic strategies include seatbelts in vehicles, helmets while riding, monitored school street crossings, high visibility clothing, protective gear in sports, and avoiding reckless behavior [10]. The large numbers documented by Fazel and colleagues (amounting to over 9% of the total cohort experiencing at least one concussion before adulthood) indicate that basic strategies need more attention. The solution is not to trivialize concussions in a misleading stereotype of bravado; neither is it to pursue a risk-free lifestyle because excess sedentary activity can also contribute to poor functioning during adulthood. The solution is prevention [11].

A great lingering uncertainty is on the treatment of patients when prevention fails and a concussion occurs. Most guidelines recommend rest for recovery and to avoid a second injury [12]. The right time to return to work or play is unknown, cannot be gauged by available biomarkers or imaging, and must be individualized. Symptomatic care for headaches, dizziness, depression, anxiety, photophobia, and insomnia is reasonable, despite a lack of randomized trial data. Generous sympathy and patience is also justified since patients can have difficulty adjusting to an invisible disease. No physician can cure a concussion, yet diligent care can stop an injury from becoming worse and may ultimately help maintain mental health and prevent adverse psychosocial outcomes.

## References

[1] Cyr A, Cyr LO, Cyr C. Acquired growth hormone deficiency with hyponodatropic hypogonadism in a subject with repeated head trauma, or Tintin goes to the neurologist. CMAJ 2004;171:1433–34. 15583175 10.1503/cmaj.1041405 PMC534570

[2] Fralick M, Thiruchelvam D, Tien HC, Redelmeier DA. Risk of suicide after a concussion. CMAJ. 2016;188(7):497–504. 10.1503/cmaj.150790 26858348 PMC4835278

[3] Sariaslan A, Sharp DJ, D’Onofrio BM, Larrson H, Fazel S. Long-term outcomes associated with traumatic brain injury in childhood and adolescence: A nationwide Swedish cohort study of a wide range of medical and social outcomes. PLoS Med. 2016; 13(8):1002103 10.1371/journal.pmed.1002103 PMC4995002 27552147

[4] Ojo JO, Mouzon BC, Crawford F. Repetitive head trauma, chronic traumatic encephalopathy and tau: Challenges in translating from mice to men. Exp Neurol. 2016;275 Pt 3:389–404. 10.1016/j.expneurol.2015.06.003 26054886

[5] Blennow K, Hardy J, Zetterberg H. The neuropathology and neurobiology of traumatic brain injury. Neuron. 2012;76(5):886–99. 10.1016/j.neuron.2012.11.021 23217738

[6] Walberg HJ, Tsai S. Matthew effects in education. American Educational Research Journal. 1983;20(3):359–373.

[7] Beadle EJ, Ownsworth T, Fleming J, Shum DJ. The impact of traumatic brain injury on self-identity: a systematic review of the evidence for self-concept changes. Head Trauma Rehabil. 2016;31(2):E12–25. 10.1097/HTR.0000000000000158 26098262

[8] Redelmeier DA, Tan SH, Booth GL. The treatment of unrelated disorders in patients with chronic medical diseases. N Engl J Med. 1998;338(21):1516–20. 9593791 10.1056/NEJM199805213382106

[9] Chambers J, Cohen SS, Hemminger L, Prall JA, Nichols JS. Mild traumatic brain injuries in low-risk trauma patients. J Trauma. 1996;41(6):976–80. 8970549 10.1097/00005373-199612000-00006

[10] World Health Organization. Preventing injuries and violence: a guide for ministries of health. Geneva: World Health Organization, 2007.

[11] Redelmeier DA, McLellan BA. Modern medicine is neglecting road traffic crashes. PLoS Med. 2013;10(6):e1001463 10.1371/journal.pmed.1001463 23776413 PMC3678998

[12] McCrory P, Meeuwisse WH, Aubry M, Cantu B, Dvorák J, Echemendia RJ, et al Consensus statement on concussion in sport: the 4th International Conference on Concussion in Sport held in Zurich. Br J Sports Med. 2013;47(5):250–8. 10.1136/bjsports-2013-092313 23479479

